# Contributions of SGK3 to transporter-related diseases

**DOI:** 10.3389/fcell.2022.1007924

**Published:** 2022-12-01

**Authors:** Qian-Qian Liao, Qing-Qing Dong, Hui Zhang, Hua-Pan Shu, Yu-Chi Tu, Li-Jun Yao

**Affiliations:** ^1^ Department of Nephrology, Tongji Medical College, Union Hospital, Huazhong University of Science and Technology, Wuhan, China; ^2^ Department of Nephrology, The Second Affiliated Hospital, Chongqing Medical University, Chongqing, China

**Keywords:** serum- and glucocorticoid-induced kinase, SGK3, channel, substance metabolism, substance transport

## Abstract

Serum- and glucocorticoid-induced kinase 3 (SGK3), which is ubiquitously expressed in mammals, is regulated by estrogens and androgens. SGK3 is activated by insulin and growth factors through signaling pathways involving phosphatidylinositol-3-kinase (PI3K), 3-phosphoinositide-dependent kinase-1 (PDK-1), and mammalian target of rapamycin complex 2 (mTORC2). Activated SGK3 can activate ion channels (TRPV5/6, SOC, Kv1.3, Kv1.5, Kv7.1, BKCa, Kir2.1, Kir2.2, ENaC, Nav1.5, ClC-2, and ClC Ka), carriers and receptors (Npt2a, Npt2b, NHE3, GluR1, GluR6, SN1, EAAT1, EAAT2, EAAT4, EAAT5, SGLT1, SLC1A5, SLC6A19, SLC6A8, and NaDC1), and Na^+^/K^+^-ATPase, promoting the transportation of calcium, phosphorus, sodium, glucose, and neutral amino acids in the kidney and intestine, the absorption of potassium and neutral amino acids in the renal tubules, the transportation of glutamate and glutamine in the nervous system, and the transportation of creatine. SGK3-sensitive transporters contribute to a variety of physiological and pathophysiological processes, such as maintaining calcium and phosphorus homeostasis, hydro-salinity balance and acid-base balance, cell proliferation, muscle action potential, cardiac and neural electrophysiological disturbances, bone density, intestinal nutrition absorption, immune function, and multiple substance metabolism. These processes are related to kidney stones, hypophosphorous rickets, multiple syndromes, arrhythmia, hypertension, heart failure, epilepsy, Alzheimer’s disease, amyotrophic lateral sclerosis, glaucoma, ataxia idiopathic deafness, and other diseases.

## 1 Introduction

Serum- and glucocorticoid-induced kinase (SGK), the AGC family of serine/threonine protein kinase, which is similar to the protein kinase B (PKB)/AKT family in exhibiting structure and sequence, is widely distributed in various tissues and organs of mammals, involving the regulation of substance transportation, hormone release, neuroexcitability, cell proliferation, and apoptosis (Lang et al., 2006a). The SGK family consists of three highly homologous mammalian isoforms, SGK1, SGK2, and SGK3, which are, respectively, encoded by three different genes (Lang et al., 2006a), but the catalytic domains of SGK3 and SGK2 have up to 80% amino acid sequence homology with SGK1 (Kobayashi and DeakM.MorriceN.Cohen, 1999). Although they are similar in function, there are differences. In this brief review, we focus on the distribution and regulation of SGK3, as well as the contribution of SGK3 to transporter-related diseases.

### 1.1 Structure and distribution of serum- and glucocorticoid-induced kinase-3

The primary structure of SGK3, known as cytokine-independent survival kinase (CISK), consists of a Phox-homology domain (PX) in the N terminus, a kinase domain (comprising Thr-320), and a protein kinase AGC C-terminal domain/hydrophobic motif (HM) (comprising Ser-486) (Xing et al., 2004). The PX domain is a phosphoinositide-binding domain, which can target the subcellular localization of SGK3 to membranes, especially early endosomes. SGK3 is distributed in almost all mammalian tissues and cell lines, including the heart, brain, lungs, skin, liver, pancreas, skeletal muscle, and kidney (Kobayashi and DeakM.MorriceN.Cohen, 1999; Yao et al., 2011). At the subcellular level, SGK3 is diffusely expressed throughout cells, covering the nucleus, cytoplasm, endosome, and plasma membrane (Lang et al., 2006a; He et al., 2011; Bago et al., 2014).

### 1.2 Regulatory mechanisms of serum- and glucocorticoid-induced kinase-3

To become functional, SGK3 is activated by phosphorylation through PI3K, PDK-1, and mTORC2 signaling pathways (Bago et al., 2014; Gasser et al., 2014; Chen et al., 2018). Insulin, growth factors (EGF, IGF1, FGF, and TPA), and oncogenic Ras can also activate SGK3 through the PI3K pathway (Malik et al., 2018). The PX domain of SGK3 binds phosphatidylinositol-3-phosphate (PI (3) P), which is activated by PI3K and targets SGK3 to the early endosome. Like other members of the AGC kinases, SGK3 is activated following the phosphorylation of its C-terminal/HM Ser-486 residue by mTORC2 and kinase domain T-loop Thr-320 residues by PDK-1 (Tessier and Woodgett, 2006; Bago et al., 2014). Unlike SGK1, although SGK3 belongs to serum- and glucocorticoid-induced kinase family, its expression is not regulated by serums and glucocorticoids (Kobayashi and DeakM.MorriceN.Cohen, 1999; Naray-Fejes-Toth et al., 2004). On the contrary, estrogen (Wang et al., 2011), androgen (Wang et al., 2014), insulin (Malik et al., 2018), and other hormones participate in the regulation of SGK3.

Numerous studies have demonstrated that SGK3 regulates downstream targets in cell survival, including B-cell lymphoma-extra-large (Bcl-xl), Bcl-2-associated death promoter (BAD), glycogen synthase kinase-3 (GSK3), forkhead family of transcription factors (FKHRs), and Flightless-1 (Fli-I) (Liu et al., 2000; Xu et al., 2009; Liu et al., 2012). Mainly, depending on GSK3-β, tuberous sclerosis factor 2 (TSC2), and proline-rich AKT substrate of 40 kDa (PRAS40), SGK3 leads to cell growth and proliferation (Bruhn et al., 2013). SGK3 also induces cell migration by downregulating the chemokine (C-X-C) receptor type-4 (CXCR4) through strong interaction and colocalization with ubiquitin ligase AIP4 (atrophin-1-interacting protein 4) (Slagsvold et al., 2006). In addition, the ubiquitin E3 ligase Nedd4-2 (neural precursor cell expressed developmentally down-regulated protein 4 subtype 2) is an important downstream target of SGK3 and has been shown to mediate the ubiquitination degradation process of various proteins (Yuan et al., 2018). In short, the activation of SGK3 plays an important role in cell proliferation, growth, survival, migration, and protein degradation (Hou et al., 2015).

### 1.3 Transporter-related diseases

Transmembrane transport of substances is widely involved in physiological and pathophysiological processes and is responsible for the transport of ions and small- and medium-size molecules. We can distinguish few types of transmembrane transports: active transport, passive transport, exocytosis, and endocytosis. The active transport is the energy-consuming process achieved by ATP hydrolysis including Ca^2+^-ATPase, Na^+^/K^+^-ATPase, and H^+^/K^+^-ATPase. The passive transport does not require energy including simple diffusion, facilitated diffusion, and osmosis, among which facilitated diffusion requires the participation of carriers, ion channels, and transporters. Ion channels, mainly contained voltage-gated channels and ligand-gated channels, are the primary mediators of muscle cell electrical signals and neuronal excitability including Na^+^, K^+^, Ca^2+^, and Cl^−^ channels (Kulbacka et al., 2017). The transmembrane transport involves complex mechanisms and is decisive for cell biological functions. This process is determined by many factors, such as the internal and external environment of cells, transporters, carriers, and ion channels and the change in any link will cause the occurrence of diseases. For example, sodium and potassium ion channels are associated with many diseases, such as epilepsy, arrhythmias, and hypertension (Kulbacka et al., 2017). Aquaporins (AQPs) are related to congestive heart failure, hypertension, glaucoma, and other diseases (Julita et al., 2017).

### 1.4 Role of serum**-** and glucocorticoid-induced kinase-3 in transporter-related diseases

#### 1.4.1 Serum**-** and glucocorticoid-induced kinase-3 and calcium–phosphorus metabolism

**FIGURE 1:**
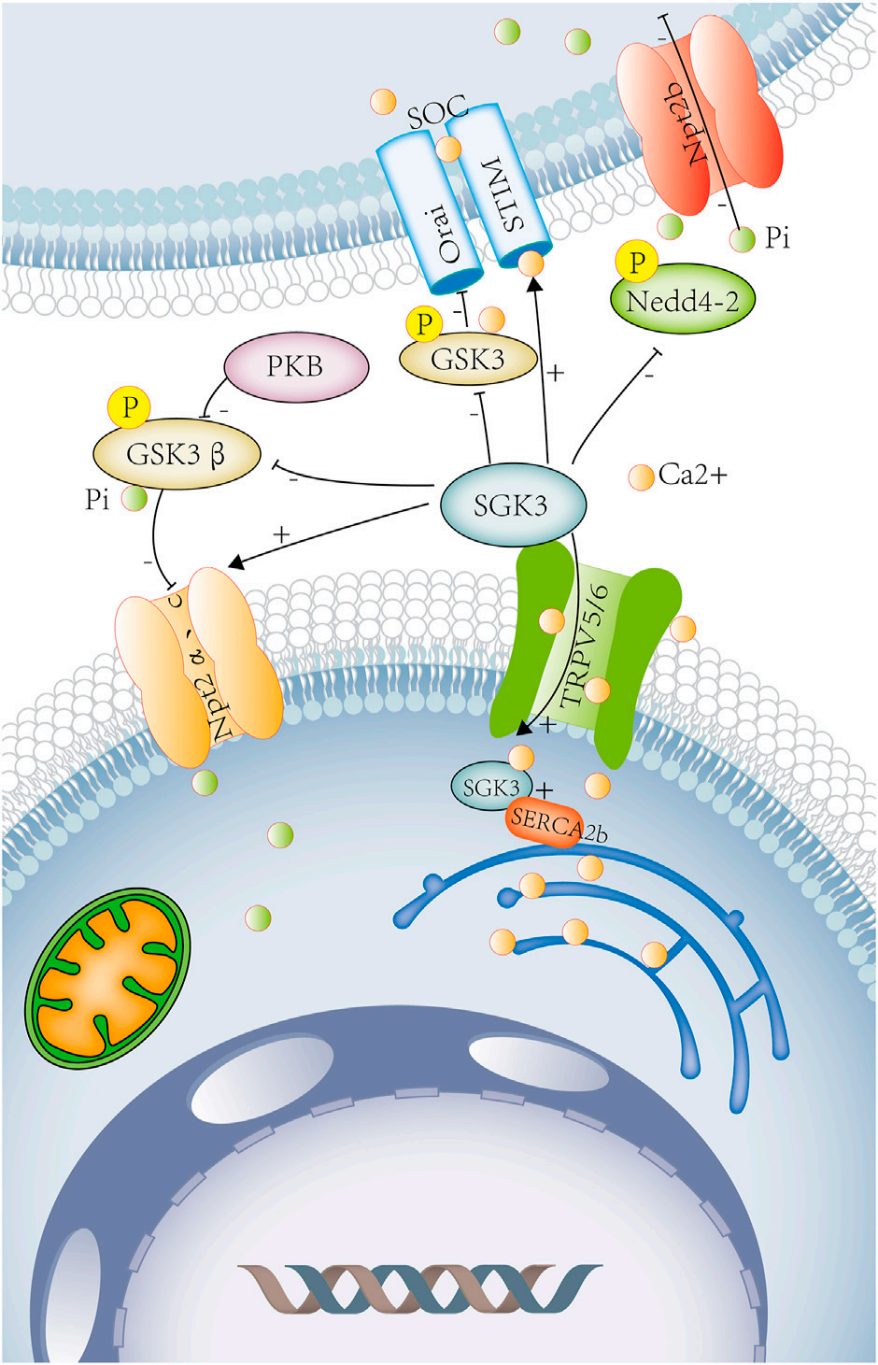
SGK3 and calcium–phosphorus metabolism. Calcium and phosphorus metabolism are closely related to intestinal absorption, renal excretion, and bone remodeling. SGK3 leads to calcium influx by increasing the number of calcium channels, TRPV5 and TRPV6, on the renal epithelial cell membrane and the brush-border membrane of intestinal epithelial cells. SGK3 affects the SOC channel by rising STIM2 and promotes the entry of Ca^2+^ into DC. It can also regulate the activity of SOC and bone mineral density by phosphorylating GSK3. SGK3 maintains ENR Ca^2+^ homeostasis by interacting with SERCA2b. SGK3 can directly regulate the activity of Npt2a. SGK3 can also phosphorylate and inhibit GSK3β activity through the PKB/SGK pathway, thereby up-regulating the activity of Npt2a. SGK3 can directly phosphorylate and inhibit the activity of Nedd4-2 and reduce the inhibition of the Npt2b activity by Nedd4-2. SGK3, serum/glucocorticoid-regulated kinase family member 3. TRPV5, transient receptor potential cation channel subfamily V member 5. TRPV6, transient receptor potential cation channel subfamily V member 6. SOC, store-operated channels. STIM2, stromal interaction molecule 2. ORAI, calcium release-activated calcium modulator 1. DC, dendritic cells. GSK3, glycogen synthase kinase-3. SERCA2b (ATP2a2) ATPase, Ca^+^ transporting, cardiac muscle, slow twitch 2. NPT2a, sodium-dependent phosphate transport protein. 2A.NPT2b, solute carrier family 34 member. 2a.GSK3β, glycogen synthase kinase 3 beta. PKB, rac-beta serine/threonine protein kinase. Orange circles represent calcium ions. Green circles represent phosphorus ions.

The strict control of calcium–phosphorus metabolism balance is the key to maintaining normal physiological functions. In skeletal tissues, calcium–phosphorus metabolism cooperates with osteoblasts and extracellular matrices to promote physiological mineralization. In soft tissues, it can prevent calcium-phosphate complexes from pathological or ectopic mineralization (Kirsch, 2006). Normally, calcium and phosphate that enter bodies through diet are mainly absorbed in the upper part of the small intestine and excreted through kidneys, intestines, and sweat glands. Thus, calcium–phosphorus metabolism is closely related to intestinal absorption, renal excretion, and bone remodeling in bodies (Peacock, 2010), depending on a variety of hormones, including parathyroid hormone (PTH) (Potts and Gardella, 2007), serum-ionized calcium and phosphate (Brown, 2007), and 1.25 (OH) 2D3 (Jurutka et al., 2001). In addition, the fibroblast growth factor-23 (FGF-23) and the FGF/Klotho-receptor complex reduce the reabsorption of phosphate by affecting sodium-dependent phosphate transport protein IIa (Npt2a) and inhibit the production of vitamin D3 by impairing 1-α hydroxylation of 25-hydroxyvitamin D, thus participating in calcium–phosphorus metabolism (Hruska et al., 2008; Liu et al., 2016) (Figure 1).

##### 1.4.1.1 Renal calcium–phosphorus metabolism

The kidney is the main site of calcium and phosphate excretion. The serum calcium filtered from the glomerulus must be reabsorbed in more than 98% along nephrons, involving the proximal tubules, thick ascending limbs of Henle’s loops, distal tubules, and collecting ducts. On the apical membrane of the distal tubular epithelium, calcium passively enter into the epithelial cells by epithelial Ca^2+^ channels (TRPV5 or TRPV6) and binds to vitamin D3-sensitive calcium-binding proteins (calbindin-D28K). (Figure 1) On the basolateral membrane, calcium is actively extruded *via* the Na^+^/Ca^2+^ exchanger (NCX1) and Ca^2+^-ATPase (Hoenderop et al., 2000). (Figure 1) The epithelial Ca^2+^ channels, TRPV5 and TRPV6, play an important role in the renal and intestinal calcium absorption. It has been found in *xenopus* oocytes that SGK3 can significantly increase TRPV5 activity and the uptake of Ca^2+^, inducing calcium currents (ICa), an effect requiring the presence of PDZ domains of NHE regulating factors NHERF2 (Embark et al., 2004a) (Figure 1). In *Xenopus* oocytes, the expression of SGK3 together with TRPV6 resulted in significant further stimulation of calcium currents (ICa) but this process does not depend on PDZ domains of NHERF2 (Böhmer et al., 2007). (Figure 1) Therefore, SGK3 induces Ca^2+^ influx by increasing the number of calcium channels TRPV5/6 on renal epithelial cell membranes, which participates in the reabsorption of calcium in the kidney, and maintains calcium homeostasis.

The kidney plays a key role in the regulation of phosphate. About 70%–80% of the phosphate filtered by the glomerulus is reabsorbed in the proximal tubule of the kidney, which is mainly mediated by Npt2a and followed by Npt2c, Pit-2 (type III sodium-dependent phosphate transporter) (Lederer, 2014; Vervloet et al., 2017). (Figure 1) The study found that compared with SGK3 wild-type mice, SGK3 knockout mice had increased urinary phosphate excretion and decreased plasma FGF23, 1.25 (OH) 2D3. The expression of SGK3 together with Npt2a in *Xenopus* oocytes could significantly enhance phosphate-induced currents and the transport of renal tubular phosphate. Therefore, SGK3 is involved in the transport of renal tubular phosphate by regulating the activity of Npt2a (Bhandaru et al., 2011). SGK3 could also phosphorylate and inhibit the activity of GSK3 through the PKB/SGK pathway by subsequently increasing renal Npt2a activity and reducing renal calcium and phosphate loss (Foller et al., 2011). (Figure 1) FGF23 is secreted by osteoblasts to regulate homeostasis of phosphate, vitamin D, and bone mineralization, which can inhibit renal tubular type II sodium-dependent phosphate transporters (Npt2a and Npt2c), reducing renal phosphate reabsorption, and promoting phosphate excretion (Baum et al., 2005; Ide et al., 2018). (Figure 1) As previously described, plasma FGF23 was reduced in SGK3 knockout mice. SGK3 may participate in renal phosphate reabsorption through FGF23 (Figure 1).

##### 1.4.1.2 Intestinal calcium–phosphorus metabolism

In the intestine, the absorption of calcium and phosphate is codependent, regulated by a variety of hormones, including 1.25 (OH) 2D3, glucocorticoid, growth hormone, and IGF-1. Among them, 1.25 (OH) 2D3 is a major factor affecting intestinal calcium and phosphate transportation, which increases intestinal calcium absorption by regulating the intestinal epithelial calcium channel TRPV6 and calcium-binding protein D9k (Christakos, 2012). In SGK3 knockout mice, plasma FGF23, 1.25 (OH) 2D3 was reduced (Bhandaru et al., 2011), thereby affecting intestinal calcium absorption. SGK3 can also directly regulate epithelial Ca^2+^ channels TRPV5 and TRPV6 on the brush-border membrane of intestinal epithelial cells and participate in intestinal calcium absorption (Embark et al., 2004a; Böhmer et al., 2007) (Figure 1).

The intestinal phosphate is absorbed through the following two pathways: passive transport (cell-tight junction) and active transport (mainly through sodium-dependent phosphate transporter Npt2b), and approximately 50% of the intestinal phosphate is absorbed and transported by the active pathway (Lederer, 2014; Hernando and Wagner, 2018). In *Xenopus* oocytes, co-expression of Nedd4-2 and Npt2b could attenuate Npt2b activity, while the effect reversed by the additional co-expression of SGK3 could directly phosphorylate and inhibit Nedd4-2 activity. Therefore, SGK3 can regulate the activity of Npt2b and participate in the absorption of intestinal phosphate by phosphorylating Nedd4-2 (Palmada et al., 2004a). (Figure 1) The plasma levels of 1.25(OH) 2D3 were reduced in SGK3 knockout mice (Bhandaru et al., 2011), and another study showed that 1.25 (OH) 2D3-induced increase in intestinal phosphate absorption is mediated by Npt2b (Xu et al., 2002). Intraperitoneal injection of 1.25 (OH) 2D3 and intragastric administration of 33P-labeled phosphate in rats can significantly increase plasma 33Pi levels, so 1.25 (OH) 2D3 could increase the intestinal transport of phosphate (Williams and DeLuca, 2007). Therefore, SGK3 may participate in intestinal phosphate absorption through 1.25 (OH) 2D3.

##### 1.4.1.3 Bone calcium–phosphorus metabolism

Hereditary hypophosphatemic rickets (HR) is a group of renal phosphate wasting disorders due to genetic mutations. A novel mutation in the SGK3 gene was found in HR patients (Cebeci et al., 2020). SGK3 gene mutation makes the loss of the Thr-320 phosphorylation site and the disruption of the protein tertiary structure, resulting in increased urine phosphate, decreased serum phosphate, and bone deformity (Cebeci et al., 2020). In Bhandaru’s study, it was found that bone density of SGK3 knockout mice decreased, which may be related to the decrease in FGF23, 1.25 (OH) 2D3 in the plasma of mice after the SGK3 knockout (Bhandaru et al., 2011). Compared with GSK3 wt mice, the bone density of GSK3 ki mice is significantly reduced, and SGK3 could inhibit the activity of GSK3 through the PKB/SGK pathway to regulate bone density (Foller et al., 2011) (Figure 1).

##### 1.4.1.4 Immune system calcium metabolism

Dendritic cells (DCs) are antigen-presenting cells that participate in both innate and adaptive immunities. Ca^2+^ signaling is involved in DC activation, maturation, migration, and the formation of immunological synapses with T cells (Shumilina et al., 2011). Ca^2+^ enters the DC partly accomplished by store-operated Ca^2+^ (SOC) channels, which consists of the plasma membrane Ca^2+^ channel Orai and the endoplasmic reticulum (ER) Ca^2+^ sensing subunit STIM. SGK3 could up-regulate the expression of STIM2, affecting the activity of SOC channels and DC migration, but the maturation and phagocytic capacity of DC is not affected by SGK3 (Schmid et al., 2012). Another study found that PKB/SGK-dependent GSK3 phosphorylation participated in the regulation of Ca^2+^ signaling in DCs, preventing DC maturation (Russo et al., 2013). Compared with DCs isolated from GSK3WT mice, DCs from GSK3KI mice had significantly reduced the levels of GSK3 phosphorylation and the expression of Orai1 and STIM2, the main components of the SOC channel, while the phosphorylation of GSK3 depends on the regulation of PBK/AKT and SGK. As shown earlier, SGK3 can regulate the release of Ca^2+^ and the activity of calcium channels (SOC) in DCs by phosphorylating GSK3 (Schmid et al., 2014) (Figure 1).

Mast cells play a decisive role in immunoglobulin E (IgE)-dependent allergic reactions, and the influx of extracellular calcium is the key to this process (Bradding et al., 2003). *In vitro*, mast cells were isolated from the bone marrow of sgk3^−/−^ and sgk3^+/+^ mice. In sgk3^−/−^ mast cells, after stimulation with IgE and related antigens, Ca^2+^ entry, Ca^2+^-activated K^+^ channel activity, and degranulation was significantly reduced. *In vivo*, in sgk3^−/−^ mice, the serum histamine levels measured 30 min after the allergic reaction induced by intraperitoneal injection of anti-DNP IgE and DNP-HSA antigen was significantly lower than that in sgk3^+/+^ mice. The measured drop in the body temperature of sgk3^−/−^ mice following antigen treatment was lower than that of sgk3^+/+^ mice, so the systemic allergic reaction of sgk3^−/−^ mice was weakened. Therefore, SGK3 affects the immune function of mast cells by regulating antigen-stimulated Ca^2+^ entry and degranulation (Zemtsova et al., 2010) (Figure 1).

##### 1.4.1.5 Endoplasmic reticulum calcium metabolism

The endoplasmic reticulum (EnR), an organelle, is the major site for protein folding and biosynthesis, and it is also a storage site for intracellular calcium. The Ca^2+^ pump sarcoplasmic/EnR calcium ATPase (SERCA) is a key enzyme that maintains high concentrations of Ca^2+^ in EnR and participates in the biosynthesis function of EnR. Studies have shown that SGK3 maintained EnR Ca^2+^ homeostasis through interaction with SERCA2b, thereby preventing it from excessive EnR stress (Wang et al., 2017) (Figure 1).

Taken together, SGK3 can regulate calcium–phosphorus metabolism through calcium channels TRPV5/6, SOC, and sodium-dependent phosphate transporter (Npt2a/2b) (Figure 1). Disorders of calcium-phosphorus metabolism can lead to various pathological changes, such as kidney stones, bone diseases, ectopic calcification, cardiovascular diseases, and abnormal immune functions (Taylor and Bushinsky, 2009).

In SGK3 and calcium–phosphorus metabolism, SGK3 induces calcium current, promotes calcium uptake, increases the absorption of calcium by renal epithelial cells and intestinal tract, and maintains calcium homeostasis by increasing the activity of TRPV5 or TRPV6. SGK3 can also affect the activity of SOC channels and DC migration by up-regulating STIM2 and is related to the immune function of mast cells, EnR Ca^2+^homeostasis. SGK3 can inactivate GSK3 and Nedd4-2 by phosphorylating them, thereby increasing the activity of Npt2a and Npt2b, enhancing the phosphate-induced current, promoting the renal tubular phosphate transport, intestinal phosphate absorption, and regulating bone mineral density (Table 1).

**TABLE 1:** SGK3 and calcium–phosphorus transport.

Cell type	Target	Result	Related disease
Renal epithelial cells (the distal tubular epithelium and the basolateral membrane)	TRPV5 or TRPV6	SGK3 induces Ca^2+^ influx by increasing the number of calcium channels TRPV5/6 , which participates in the reabsorption of calcium in the kidney, and maintains calcium homeostasis	Kidney stones
Xenopus oocytes	TRPV5 or TRPV6	SGK3 can significantly increase TRPV5 activity and the uptake of Ca^2+^, inducing calcium currents (ICa)	
Proximal tubular cells and xenopus oocytes	Npt2a and GSK3	1. Expression of SGK3 together with Npt2a could significantly enhance phosphate-induced currents and the transport of renal tubular phosphate	Kidney stones and hereditary hypophosphataemic rickets (HR)
		2. SGK3 could also phosphorylate and inhibit the activity of GSK3 through the PKB/SGK pathway, subsequently increasing renal Npt2a activity and reducing renal phosphate loss	
		3. It was found that the bone density of SGK3 knockout mice decreased. 4. Compared with GSK3 wt mice, the bone density of GSK3 ki mice is significantly reduced, and SGK3 could inhibit the activity of GSK3 through the PKB/SGK pathway to regulate bone density	
Intestinal epithelial cells	TRPV5 and TRPV6	Increases intestinal calcium absorption	
Xenopus oocytes	Npt2b and Nedd4-2	1. Co-expression of Nedd4-2 and Npt2b could attenuate the Npt2b activity, while an effect reversed by SGK3, which can directly phosphorylate and inhibit Nedd4-2 activity	
		2. Plasma levels of 1.25(OH) 2D3 were reduced in SGK3 knockout mice, and Npt2b can increase intestinal phosphate uptake induced by 1.25 (OH) 2D3	
Dendritic cells (DCs)	SOC = Orai1+STIM2	SGK3 could up-regulate the expression of STIM2, affecting the activity of SOC channels and DC migration	Abnormal immune function
Mast cells		SGK3 affects the immune function of mast cells by regulating the antigen-stimulated Ca^2+^ entry and degranulation	Abnormal immune function
Endoplasmic reticulum (EnR) HEK 293T	SERCA2b	SGK3 maintained EnR Ca^2+^ homeostasis through interaction with SERCA2b, thereby preventing it from excessive EnR stress	

#### 1.4.2 Serum**-** and glucocorticoid-induced kinase-3 and potassium transport

Potassium transport is closely related to potassium channels, potassium transporters, and Na^+^/K^+^-ATPase. The subtypes of potassium channels mainly include voltage-gated K^+^ channels (Kv), inwardly rectifying K^+^ channels (Kir), two-P K^+^ channels (K2p), and Ca2^+^-activated K^+^ channels (KCa). Potassium ions maintain cell membrane potential, participating in muscle-cell electrical signals and neuronal excitability and are associated with a variety of nervous and cardiovascular system diseases (Humphries and Dart, 2015).

##### 1.4.2.1 Cardiovascular system potassium transport

**FIGURE 2:**
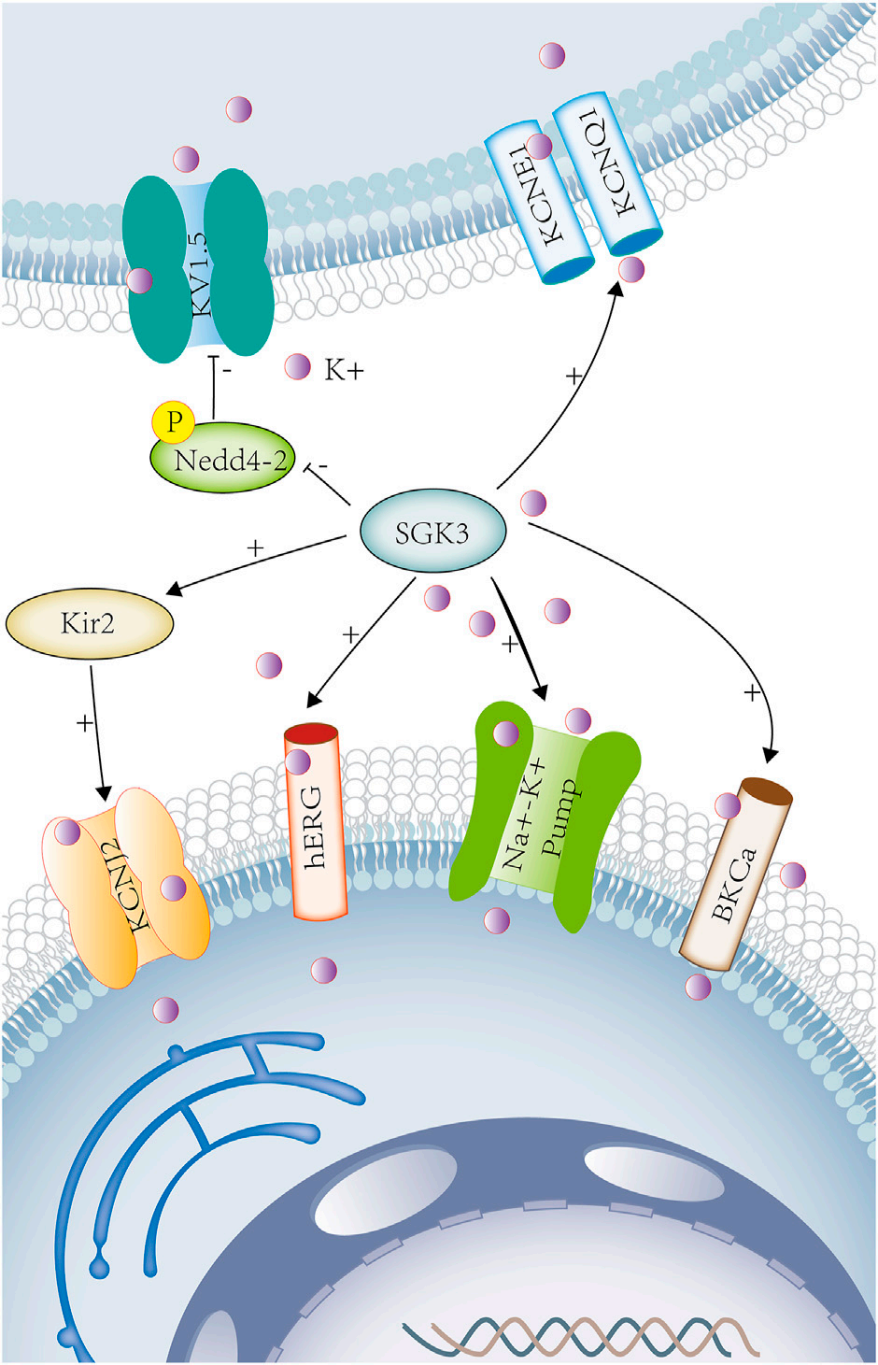
SGK3 and potassium transport. Potassium is involved in maintaining cellular electrical signals and cell excitability and is associated with a variety of neurological and cardiovascular diseases. SGK3 can enhance Kir2-mediated inward current. Kir2 gene encodes potassium voltage-gated channel subfamily J member 2 (KCNJ2). Sgk3 can up-regulate the activity and abundance of HERG. HERG (human ether-related gene-1, or kv11.1) belongs to the fast activation delay rectifier K^+^ current (IKr). Co-expression of SGK3 and KCNE1/KCNQ1 up-regulates KCNE1/KCNQ1-induced delayed potassium rectifier current (IKs). SGK3 can regulate KV15 by phosphorylating Nedd4-2. SGK3 can up-regulate Na^+^/K^+^-ATPase activity. The co-expression of BKCa and SGK3 can increase the activity of BKCa.SGK3 serum/glucocorticoid-regulated kinase family member 3. KIR2, inwardly rectifying potassium channel 2. KCNJ2, potassium inwardly rectifying channel subfamily J member 2. HERG, potassium voltage-gated channel subfamily H member 2. KCNE1, potassium voltage-gated channel subfamily E regulatory subunit 1. KCNQ1, potassium voltage-gated channel subfamily Q member 1 KV1.5(KCNA5), potassium voltage-gated channel subfamily A member 5. Na^+^-K^+^ ATPASE, ATPase Na^+^/K^+^ transporting subunit. BKCA, potassium large conductance calcium-activated channel. Purple circles represent potassium ion.

Potassium ions are involved in the repolarization phase of myocardial action potential and are closely related to a variety of arrhythmias, which can also be used as targets for the treatment of certain arrhythmias (Humphries and Dart, 2015). In *Xenopus* oocytes, SGK3 could enhance Kir2.1-mediated currents and the channel protein abundance in the cell membrane (Munoz et al., 2014) (Figure 2). The loss-of-function mutation of potassium voltage-gated channel subfamily J member 2 (KCNJ2), the encoding gene of Kir2.1, could lead to Andersen–Tawil syndrome accompanied by periodic paralysis, cardiac arrhythmia, and dysmorphic features (Seebohm et al., 2012). SGK3 could also increase Kir2.2 inward currents, and the gain-of-function mutation of KCNJ could cause atrial fibrillation (Xia et al., 2005) (Figure 2). Consequently, SGK3 may participate in the occurrence of these diseases by regulating Kir2 inward currents and protein expressions.

hERG (human ether-a-go-go-related gene 1, or Kv11.1) belongs to the rapidly activating delayed rectifier K^+^ current (IKr), which functions in conjunction with the auxiliary subunit KCNE2 (Eldstrom and Fedida, 2011). SGK3 could up-regulate the activity and abundance of hERG and change the duration of cardiac action potential. (Figure 2) The loss-of-function mutations of hERG could lead to long QT syndrome, fatal ventricular arrhythmia, or sudden death (Maier et al., 2006). KCNQ1 (a pore-forming tetramer) and KCNE1 (an auxiliary subunit) combine to form the delayed potassium rectifier current (IKS) (Larsson et al., 2018). KCNE1/KCNQ1 (Kv7.1) is widely expressed in cardiac myocytes, the stria vascularis of inner ears, renal tubules, and intestinal epithelial cells (Bleich and Warth, 2000). In *Xenopus* oocytes, the co-expression of SGK3 and KCNE1/KCNQ1 could up-regulate the KCNE1/KCNQ1-induced current, and the mutation of KCNE1/KCNQ1 led to Jervell and Lange–Nielsen syndrome, which is a disorder characterized by congenital profound sensorineural deafness, cardiac long QT syndrome, ventricular arrhythmias, and sudden cardiac death (Embark et al., 2003; Faridi et al., 2019). (Figure 2) SGK3 could also regulate the cardiac potassium channel Kv1.5 (KCNA5) by phosphorylating Nedd4-2 and participated in cardiac action potential (Ahmed et al., 2016a). (Figure 2)As previously described, potassium channels (hERG, KCNE1/KCNQ1, and Kv1.5) are involved in the repolarization phase of myocardial action potential.

Big Ca^2+^-activated K^+^ channel (BKCa) is a transmembrane protein, also known as BK, Slo1, KCa1.1, or MaxiK channel (Toro et al., 2014). It is widely expressed in vascular smooth muscle cells, cardiomyocytes, nerve cells, and skeletal muscle cells and regulates neuronal excitation, cell volume, vascular tone, and blood pressure (Ahmed et al., 2016b). The activation of BKCa channel results in increasing potassium outflow, vasodilation, and cardioprotection (Dong et al., 2016). In *Xenopus* oocytes, the co-expression of BKCa and SGK3 could increase the activity of BKCa and participate in the regulation of cell volume and neuroexcitability (Ahmed et al., 2016b). (Figure 2) The BKCa channel is associated with numerous cardiovascular diseases, such as hypertension, diabetes, metabolic syndrome, and heart failure, and is a potential therapeutic target for various cardiovascular diseases (Dong et al., 2016).

Na^+^/K^+^-ATPase is an important membrane protein that regulates the concentration of Na^+^ and K^+^ inside and outside the cell to maintain fluid and electrolyte balance in the body. In *Xenopus* oocytes, SGK3 could up-regulate Na^+^/K^+^-ATPase activity (Henke et al., 2002), which provides power for cardiac contraction and is related to obesity, diabetes, hypertension, and cardiac insufficiency (Yan et al., 2017). (Figure 2) In addition, SGK3 could also participate in the development of cardiac hypertrophy and fibrosis by regulating the phosphorylation of GSK3β (Wyatt et al., 2006).

As previously mentioned , SGK3 can regulate the potassium ion transport (Figure 2), participating in the repolarization of action potential, maintaining cell volume, and excitability, and is closely related to the occurrence and development of arrhythmia, sudden death, hypertension, obesity. and other diseases.

##### 1.4.2.2 Nervous system potassium transport

Both SGK1 and SGK3 could up-regulate the voltage-gated K^+^ channel Kv1.3 by phosphorylating Nedd4-2 (Gamper et al., 2002). (Figure 2) Potassium channels regulate neuronal excitability and membrane potential, participating in learning and memory (Kourrich et al., 2001), and are closely related to early memory loss in Alzheimer’s disease (Alkon, 1999). In *Xenopus* oocytes, SGK3 could increase the activity of big Ca^2+^-activated K^+^ channel (BKCa) (Ahmed et al., 2016b). (Figure 2) The loss of BKCa channel function can also cause neuronal hyperexcitability, which may lead to seizures (N'Gouemo, 2014). SGK3 could also regulate the activity of Na^+^/K^+^-ATPase in neurons, the osmotic pressure and transmembrane potential inside and outside neurons, and maintaining integrity and excitability of neurons, and is related to depression (Henke et al., 2002; Gamaro et al., 2014) (Figure 2).

##### 1.4.2.3 Renal potassium transport

*In vitro*, the co-expression of SGK3 and KCNE1/KCNQ1(Kv7.1) in *Xenopus* oocytes could up-regulate the activity of the potassium channel KCNE1/KCNQ1 (Embark et al., 2003). *In vivo*, KCNE1-deficient mice showed that the renal proximal tubular reabsorption function was mildly impaired because of decreased ability to maintain membrane potential in renal proximal tubules and increased urinary excretions including fluid, Na^+^, Cl^−^, and glucose (Vallon et al., 2001). Thus, SGK3 affects renal reabsorption by regulating potassium channels KCNE1/KCNQ1. (Figure 2)

In SGK3 and potassium transport, SGK3 can participate in up-regulating the activities of Kir2.1, Kir2.2, hERG, Kv7.1, BKCa, and Na^+^/K^+^- ATPase or indirectly enhance the potassium current induced by KCNA5 and Kv1.3 through the phosphorylation of Nedd4-2, participating in cardiac action potential, provide power for cardiac contraction, participate in renal proximal tubular membrane potential, maintain renal proximal tubular reabsorption function, and regulate neuronal excitability (Table 2).

**TABLE 2:** SGK3 and potassium transport.

Cell type	Target	Result	Related disease
Xenopus oocytes	Kir2.1 and Kir2.2	1. SGK3 could enhance Kir2.1-mediated currents and the channel protein abundance in the cell membrane	1. Loss-of-function mutation of KCNJ2 could lead to Andersen–Tawil syndrome accompanied by periodic paralysis, cardiac arrhythmia, and dysmorphic
		2. SGK3 could also increase Kir2.2 inward currents	2. Gain-of-function mutation of KCNJ could cause atrial fibrillation
Xenopus oocytes HEK 293	hERG	SGK3 could up-regulate the activity and abundance of hERG and change the duration of cardiac action potential	Loss-of-function mutations of hERG could lead to long QT syndrome, fatal ventricular arrhythmia, or sudden death
Xenopus oocytes	KCNE1/KCNQ1(Kv7.1)	1. Co-expression of SGK3 and KCNE1/KCNQ1 could up-regulate the KCNE1/KCNQ1-induced current	Mutation of KCNE1/KCNQ1 led to Jervell and Lange–Nielsen syndrome
		2. *In vivo*, KCNE1-deficient mice showed that the renal proximal tubular reabsorption function was mildly impaired because of decreased ability to maintain membrane potential in renal proximal tubules and increased urinary excretion including fluid, Na^+^, Cl^−^, and glucose	
Xenopus oocytes	KCNA5 (Kv1.5) and Nedd4-2	SGK3 could positively regulate the cardiac potassium channel Kv1.5 (KCNA5) by phosphorylating Nedd4-2 and participated in cardiac action potential	
Xenopus oocytes	BKCa (Big Ca^2+^-activated K^+^ channel)	Co-expression of BKCa and SGK3 could increase the activity of BKCa and participated in the regulation of cell volume and neuroexcitability	BKCa channel is associated with numerous cardiovascular diseases, such as hypertension, diabetes, metabolic syndrome, and heart failure, and the loss of BKCa channel function may also lead to overexcitation of neurons, leading to seizures
Xenopus oocytes	Na^+^/K^+^-ATPase and GSK3β	SGK3 could up-regulate the Na^+^/K^+^-ATPase activity, which provides power for the cardiac contraction	1. Related to obesity, diabetes, hypertension, depression, and cardiac insufficiency. 2. SGK3 could also participate in the development of cardiac hypertrophy and fibrosis by regulating the phosphorylation of GSK3β
Xenopus oocytes	Kv1.3 and Nedd4-2	SGK3 could up-regulate the voltage-gated K^+^ channel Kv1.3 by phosphorylating Nedd4-2	

#### 1.4.3 Serum**-** and glucocorticoid-induced kinase-3 and sodium transport

Sodium ions play an important role in the transmission of electrical signals in cells and participate in the depolarization of nerve and muscle action potentials. The abnormal structure or function of sodium channels usually leads to diseases such as failure of cardiovascular system and nervous system.

##### 1.4.3.1 Renal and lung sodium transport

**FIGURE 3:**
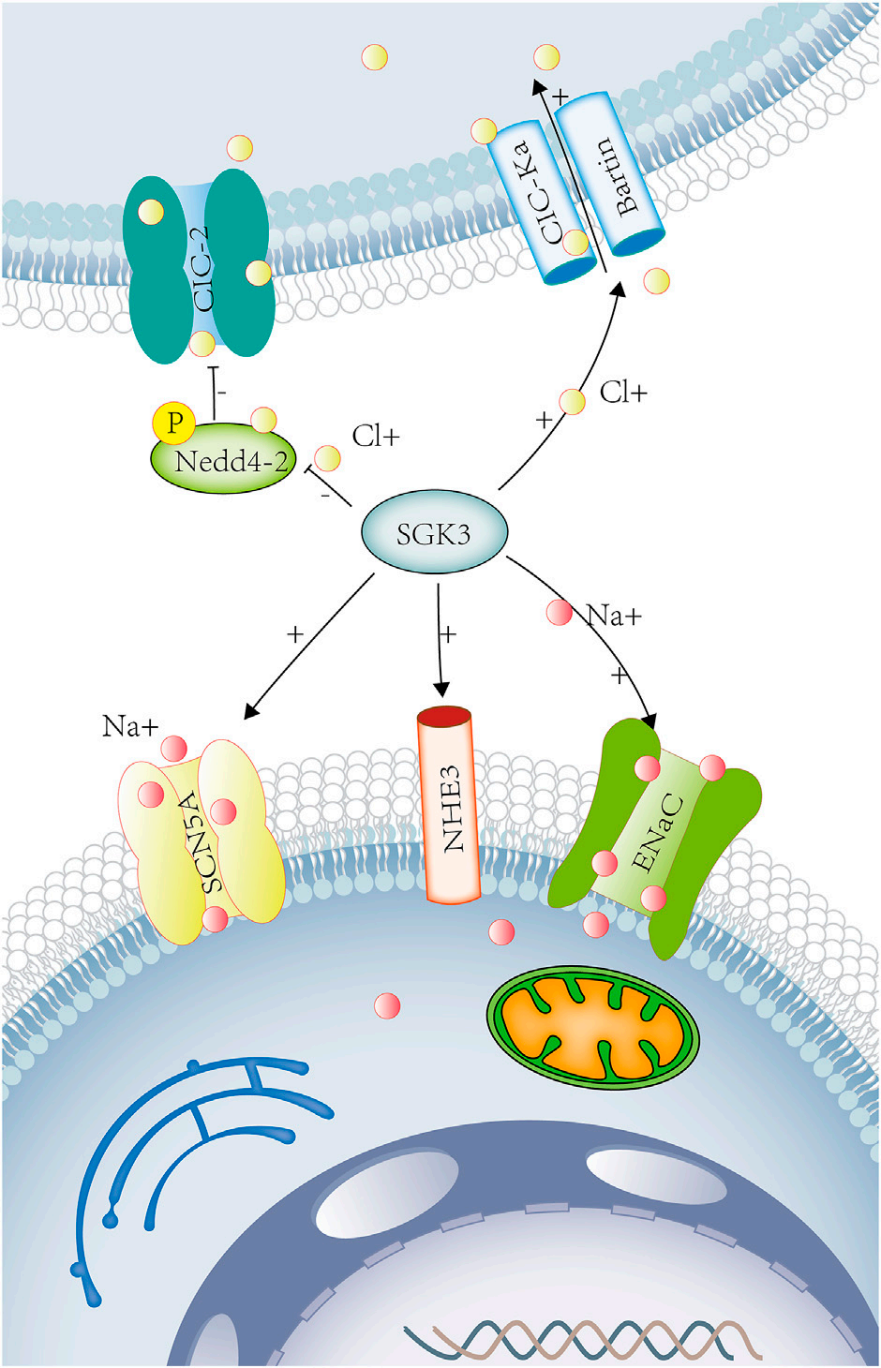
SGK3 and sodium chloride transport. Sodium ions participate in the nerve and muscle action potentials. Chloride ions participate in the regulation of neuronal excitability and cell volume. Sodium chloride maintains extracellular fluid osmotic pressure and participates in the regulation of acid-base balance in the body. SGK3 can raise the activity of ENaC. The Phox-homology (PX) domain of SGK3 can be colocated with NHE3 and participate in the acute activation of NHE3 by glucocorticoids. SGK3 can regulate myocardial sodium channel SCN5A (Nav1.5). SGK3 can raise CIC-Ka/Bartin-induced current. SGK3 indirectly regulates the activity and quantity of ClC-2 by phosphorylating Nedd4-2. SGK3, serum/glucocorticoid-regulated kinase family member 3. ENaC, sodium channel, nonvoltage-gated 1 alpha. NHE3, Na [^+^]/H [^+^] hydrogen exchanger 3. SCN5A, sodium voltage-gated channel alpha subunit 5. CIC-Ka/barttin chloride channel protein ClC-Ka/Bartin.CIC-2 chloride voltage-gated channel 2. Red circles represent sodium ions. Yellow circles represent chloride ions.

The epithelial sodium channel (ENaC) is mainly expressed in the kidney, lungs, salivary glands, and skin as well as maintaining water and salt homeostasis, which can affect the reabsorption of sodium in the kidney, regulating the epithelial surface liquid volume in the respiratory system (Hanukoglu and Hanukoglu, 2016). SGK3 could up-regulate the activity of ENaC (Figure 3) but it was not achieved by directly phosphorylating ENaC (Friedrich et al., 2003). SGK1 could indirectly regulate the activity and quantity of ENaC by phosphorylating Nedd4-2 (Debonneville et al., 2001). Therefore, SGK3 may, similar to SGK1, indirectly participate in the regulation of ENaC activity and the reabsorption of renal sodium and influences fluid balance and blood pressure (Hanukoglu and Hanukoglu, 2016).

##### 1.4.3.2 Intestinal sodium transport

Na^+^/H^+^ exchanger 3 (NHE3) is the main sodium transport protein in the intestine, which is located at the brush-border membrane of the intestinal epithelium and the endosomal compartment. The Phox-homology (PX) domain of SGK3 could be colocalized with NHE3 to recycling endosomes, participating in the acute activation of NHE3 by glucocorticoids. (Figure 3) Although SGK3 was not directly affected by glucocorticoids, the activation of SGK3 and NHE3 by glucocorticoids was dependent on the PI3K-PDK-1 pathway (He et al., 2011). Therefore, SGK3 takes part in Na^+^ reabsorption and H^+^ secretion of epithelial cells through NHE3 (Figure 3) and play a role in the sodium transport and acid-base balance.

##### 1.4.3.3 Cardiac sodium transport

The voltage-gated sodium channel 1.5 (Nav1.5 and SCN5A) is abundantly expressed in skeletal muscles and myocardium. In *Xenopus* oocytes, the co-expression of SGK3 and SCN5A could enhance the activity of SCN5A. SGK3 could regulate the cardiac sodium channel Nav1.5 (Figure 3) and the depolarization of cardiac action potential, involving in various arrhythmia diseases (Boehmer et al., 2003a).

#### 1.4.4 Serum**-** and glucocorticoid-induced kinase-3 and chloride transport

##### 1.4.4.1 Renal and inner ear chloride transport

The voltage-gated chloride channels (ClC) include ClC-1-7, ClC-Ka, and ClC-Kb, in which ClC-Ka and ClC-Kb require the co-expression of barttin to become functional (Waldegger et al., 2002), only in the kidney and stria vascularis of the inner ear. In *Xenopus* oocytes, SGK3 up-regulated ClC-Ka/barttin-induced currents, while ubiquitin ligase Nedd4-2 down-regulated ClC-Ka/barttin activity, an effect reversed by SGK3 (Embark et al., 2004b) (Figure 3). The loss of function of ClC-Ka/barttin channels results in idiopathic deafness and Bartter syndrome, which is characterized by the renal dysfunction of concentration, accompanied by nephrogenic diabetes insipidus, hyponatremia, hypokalemia, metabolic alkalosis, and hypovolemia (Stolting et al., 2014).

##### 1.4.4.2 Ubiquitous chloride transport

The chloride channel ClC-2 is widely distributed in the brain, heart, and gastrointestinal tract, involved in the regulation of neuronal excitability, chloride secretion, and cell volume. The amino acid sequence of ClC-2 contains a consensus site (Ser82) for phosphorylation by SGK3. In *Xenopus* oocytes, inhibiting the function of Ser82, SGK3 still increased the activity and number of ClC-2. SGK3 did not directly phosphorylate ClC-2 but indirectly regulated the activity and quantity of ClC-2 through the phosphorylation of Nedd4-2 (Palmada et al., 2004b) (Figure 3).

In SGK3 and sodium chloride transport, SGK3 is involved in up-regulating the activity of ENaC, NHE3, and Nav1.5, affecting the reabsorption of sodium by kidneys and intestines, regulating the depolarization of cardiac action potential, and maintaining hydro-salinity balance and acid-base balance. SGK3 indirectly regulates the activity and quantity of ClC Ka and ClC-2 through the phosphorylation of Nedd4-2, promotes the transport of chloride ions, and regulates the excitability of neurons (Table 3).

**TABLE 3:** SGK3 and sodium, chloride transport.

Cell type	Target	Result	Related disease
Xenopus oocytes	ENaC (epithelial sodium channel )	SGK3 could up-regulate the activity of ENaC. The epithelial sodium channel (ENaC) is mainly expressed in the kidney, lungs, salivary glands and skin, maintaining water and salt homeostasis, which can affect the reabsorption of sodium in the kidney, and regulating epithelial surface liquid volume in the respiratory	
Brush-border membrane of the intestinal epithelium and the endosomal compartment	NHE3 (Na^+^/H^+^ exchanger 3)	SGK3 takes part in the Na^+^ reabsorption and H^+^ secretion of epithelial cells through NHE3 and play a role in the sodium transport and acid-base balance	
Xenopus oocytes	Nav1.5, (SCN5A)	Co-expression of SGK3 and SCN5A could enhance the activity of SCN5A	Arrhythmia diseases
Xenopus oocytes	ClC-Ka and Nedd4-2	SGK3 up-regulated the ClC-Ka/barttin-induced currents, while Nedd4-2 down-regulated ClC-Ka/barttin activity, an effect reversed by SGK3	Loss of function of ClC-Ka/barttin channels results in idiopathic deafness and Bartter syndrome
Xenopus oocytes	ClC-2 and Nedd4-2	1. SGK3 could increase the activity and the number of ClC-2. SGK3 did not directly phosphorylate ClC-2 but regulated the activity and quantity of ClC-2 through the phosphorylation of Nedd4-2	
		2. ClC-2 is widely distributed in the brain, heart, and gastrointestinal tract, involving in the regulation of neuronal excitability, chloride secretion and cell volume	

#### 1.4.5 Serum**-** and glucocorticoid-induced kinase 3 and glucose and amino acid transport

##### 1.4.5.1 Renal and intestinal glucose transport

**FIGURE 4:**
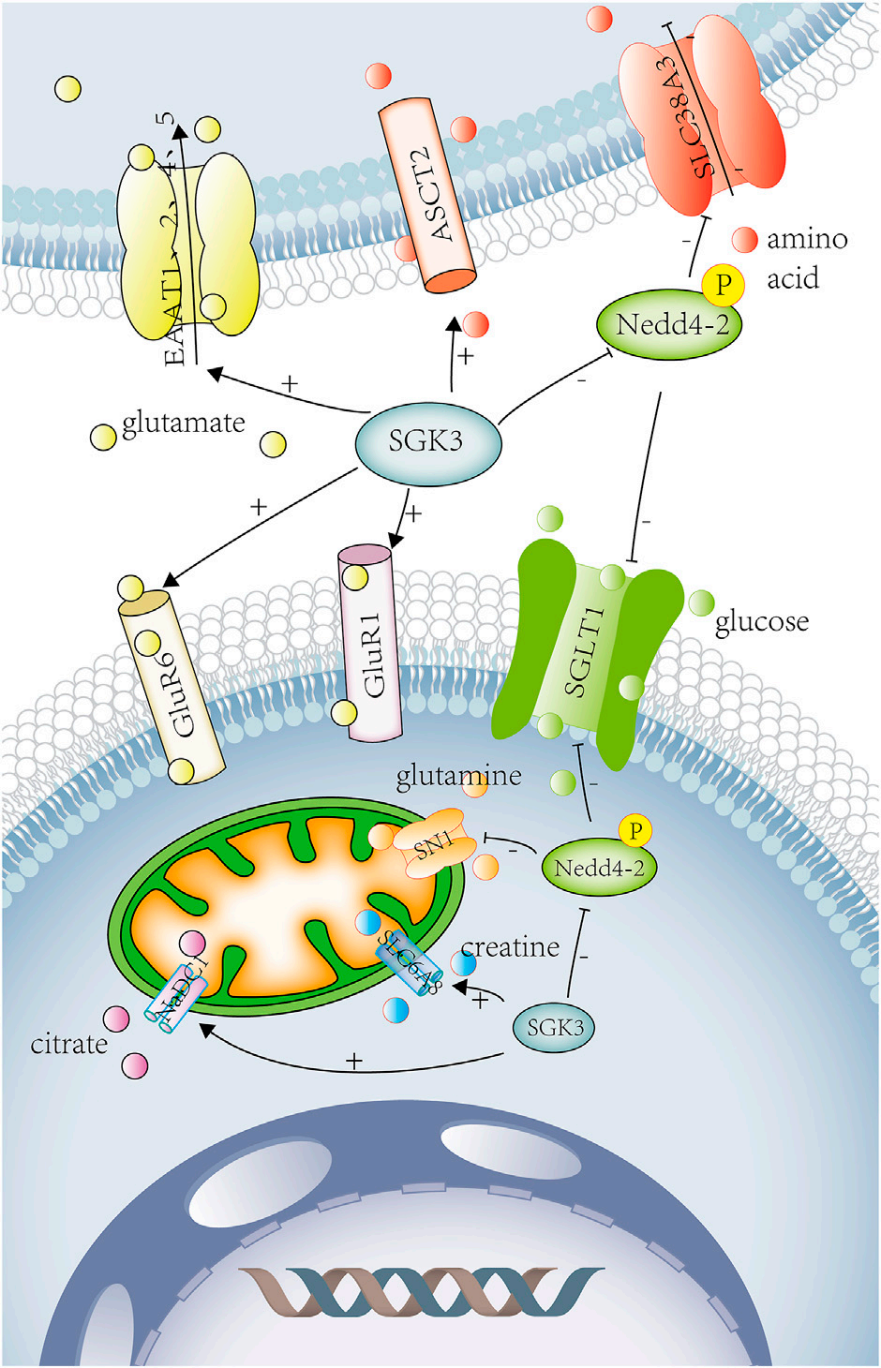
SGK3 and glucose ,amino acid ,other substance transport. SGK3 can increase SGLT1 activity and accelerate glucose absorption by phosphorylating Nedd4-2, which is related to diabetes and obesity. SGK3 can increase the expression and activity of ASCT2. SGK3 regulates the activity and expression of SLC6A19 by affecting the ubiquitin ligase Nedd4-2. SGK3 can increase the expression of glutamate receptor-1 (GluR1) in neuronal synapses and promote the glutamate transport. SGK3 can also raise the activity of GluR6. SGK3 can stimulate the expression of excitatory amino acid transporters (EAAT1, EAAT2, EAAT4, and EAAT5) and promote the uptake of glutamate. SGK3 can indirectly regulate SN1 (SLC38A3/SNAT3) activity through the phosphorylation and inhibition of Nedd4-2. SGK3 is an effective regulator of CreaT, which may regulate the uptake of creatine. SGK3 can enhance the activity of NaDC1, promote the reabsorption of citrate in renal tubules, and promote the occurrence of calcium urolithiasis. SGLT1, solute carrier family 5 member 1. Nedd4-2 E3, ubiquitin-protein ligase Nedd4. ASCT2, solute carrier family 1 member 5. SLC6A19, solute carrier family 6 member 19. GluR1, glutamate ionotropic receptor AMPA-type subunit 1. GluR6, glutamate ionotropic receptor kainate-type subunit 2. EAAT1, excitatory amino acid transporter-1. EAAT2, excitatory amino acid transporter-2. EAAT4, excitatory amino acid transporter-4. EAAT5, excitatory amino acid transporter-5. SN1, solute carrier family 38 member 3. SLC6A8(CreaT), solute carrier family 6 member 8. NaDC1, solute carrier family 13 member 2. Red circles represent amino acid. Yellow circles represent glutamate. Green circles represent glucose. Purple circle represents citrate. Orange circles represent glutamine. Blue circles represent creatine.

The sodium-glucose cotransporter-1 (SGLT1) is involved in glucose (re)absorption in the kidney and intestines. *In vitro*, SGK3 could increase SGLT1 activity by phosphorylating Nedd4-2 and accelerate glucose absorption, which is related to diabetes and obesity (Dieter et al., 2004). (Figure 4) *In vivo*, compared with SGK3 wild-type mice, the SGLT1 function and intestinal glucose transport of SGK3 knockout mice were impaired (Sandu et al., 2005).

##### 1.4.5.2 Renal and intestinal amino acid transport

SLC6A19 is a sodium-dependent neutral amino acid transporter, which covered the transport of neutral amino acids in the kidney and intestines. The amino acid sequence of SLC6A19 contains the consensus phosphorylation site 100Ser for SGK3, but SGK3 stimulated SLC6A19 activity and expression by influencing the ubiquitin ligase Nedd4-2 independently of 100Ser without modifying carrier substrate affinities (Bohmer et al., 2010). The Na^+^-dependent neutral amino acid transporter type-2 (ASCT2/SLC1A5) is widely expressed in lungs, kidneys, intestines, placenta, pancreas, and other tissues and plays an important role in the transport of neutral amino acid in the epithelial cells. SGK3 could up-regulate the expression and activity of ASCT2, thereby regulating the absorption of cellular nutrients (Palmada et al., 2005) (Figure 4).

##### 1.4.5.3 Nervous system amino acid transport

SGK3 can increase the expression of glutamate receptor-1 (GluR1) in neuronal synapses and promote the transport of glutamate. In *Xenopus* oocytes, the co-expression of SGK3 and GluR1 significantly increased GluR1-current amplitudes and protein abundance (Strutz-Seebohm et al., 2006). SGK3 could also up-regulate the expression and activity of GluR1 in murine hippocampal neurons, and the hippocampal abundance of GluR1 was significantly lower in Sgk3^−/−^ than in Sgk3^+/+^ mice (Strutz-Seebohm et al., 2005a). (Figure 4) GluR is the most important mediator of excitatory signaling in the central nervous system, and GluR1 is necessary for long-term potentiation (LTP) of hippocampal CA1 function, which participates in the retention of spatial memory (Lee et al., 2003). Therefore, SGK3 can up-regulate the GluR1 expression and activity to strengthen spatial memory. SGK3 could also up-regulate the activity of GluR6, maintaining neuronal excitability and memory consolidation (Strutz-Seebohm et al., 2005b). (Figure 4) Another study showed that SGK, as a downstream target of MAPK/ERK, could also participate in the formation of spatial memory (Lee et al., 2006). Compared with SGK3^+/+^, SGK3^−/−^ mice led to subtle behavioral defects such as reduction of locomotor activity, shorter moving distance, slower speed, and slight impairment in recognition of spatial precision (Lang et al., 2006b).

SGK3 can stimulate the expression of excitatory amino acid transporters (EAAT1, EAAT2, EAAT4, and EAAT5) (Boehmer et al., 2003b; Bohmer et al., 2004; Boehmer et al., 2005; Boehmer et al., 2006) and contribute to glutamate uptake. (Figure 4) Glutamate is the main excitatory neurotransmitter in the central nervous system. When the functions of glutamate receptors and transporters are impaired, it has indeed been shown to promote the accumulation of extracellular glutamate, resulting in neuroexcitotoxicity and ultimately cell death. Increased glutamate concentration and decreased EAAT1 expression could be seen in the epileptogenic foci of intractable temporal lobe epilepsy (TLE) (Sarac et al., 2009). The loss of EAAT function and accumulation of glutamate is also closely related to Alzheimer’s disease (AD), amyotrophic lateral sclerosis (ALS), glaucoma, and diabetic retinopathy (Boehmer et al., 2005; Magi et al., 2019). These diseases may be related to the expression of the glutamate transporter regulated by SGK3.

Glutamine transporter SN1 (SLC38A3/SNAT3) is mainly expressed in astrocytes, hepatocytes, and kidneys, coupled with Na^+^ to participate in glutamine uptake and efflux. Glutamine is a precursor of purine, pyrimidines, glucose, and protein synthesis and participates in various metabolic processes, such as the glutamine/glutamate-γ-aminobutyric acid (GABA) cycle, hepatic and renal gluconeogenesis, and hepatic ammonia detoxification. Mice deficient in SN1 can cause ataxia and disorder in amino acid homeostasis and glucose metabolism (Chan et al., 2016). SGK3 can indirectly regulate SN1 activity through phosphorylation and inhibition of Nedd4-2 (Boehmer et al., 2003c) and participate in the occurrence of the aforementioned diseases. (Figure 4)

In short, SGK3 can tune GluR1, GluR6, EAAT1, EAAT2, EAAT4, EAAT5, and SN1 (Figure 4), participating in the transport of glutamate and glutamine in the nervous system, and play a role in the (patho) physiological process of various nervous system diseases (Table 4).

**TABLE 4:** SGK3 and glucose, amino acid, and other substance transport.

Cell type	Target	Result	Related disease
Xenopus oocytes and jejunum epithelial cells	SGLT1	*In vitro*, SGK3 could increase the SGLT1 activity by phosphorylating Nedd4-2 and accelerate glucose absorption. *In vivo*, compared with SGK3 wild-type mice, the SGLT1 function and intestinal glucose transport of SGK3 knockout mice were impaired	Diabetes and obesity
Xenopus oocytes	SLC6A19 and Nedd4-2	SGK3 stimulated SLC6A19 activity and expression by influencing the ubiquitin ligase Nedd4-2 independently of 100Ser without modifying carrier substrate affinities	
Xenopus oocytes	ASCT2	SGK3 could up-regulate the expression and activity of ASCT2, thereby regulating the absorption of cellular nutrients	
Xenopus oocytes and murine hippocampal neurones	GluR1	SGK3 can up-regulate the GluR1 expression and activity in neuronal synapses and promote the transport of glutamate	
Xenopus oocytes	GluR6	SGK3 could also up-regulate the activity of GluR6, maintaining neuronal excitability and memory consolidation	
Xenopus oocytes	EAAT1, EAAT2, EAAT4, and EAAT5	SGK3 can stimulate the expression of excitatory amino acid transporters (EAAT1, EAAT2, EAAT4, and EAAT5) and contribute to glutamate uptake	Loss of EAAT function and accumulation of glutamate is closely related to intractable temporal lobe epilepsy (TLE), Alzheimer’s disease (AD), amyotrophic lateral sclerosis (ALS), glaucoma, and diabetic retinopathy
Xenopus oocytes	SN1 (SLC38A3/SNAT3) and Nedd4-2	SGK3 can reverse the inhibition of SN1 by Nedd4-2 through phosphorylation and inhibition of Nedd4-2	Mice deficient of SN1 can cause ataxia and disorders in amino acid homeostasis and glucose metabolism
Xenopus oocytes	CreaT and SLC6A8	SGK3 was a potent positive regulator of CreaT, which might regulate the creatine uptake	CreaT defect could lead to seizures, intellectual disability, and language disorders
Xenopus oocytes	NaDC1	SGK3 could enhance the activity of NaDC1, promoting renal tubular reabsorption of citrate	Promoting the occurrence of calcium urolithiasis

#### 1.4.6 Serum**-** and glucocorticoid-induced kinase 3 and other substance transport

##### 1.4.6.1 Creatine transport

Creatine transporter (CreaT, SLC6A8), a superfamily of Na^+^,Cl^−^ coupled transporters, is widely expressed in the brain, kidney, heart, skeletal muscle, and intestinal tissues, involving in the transport of various neurotransmitters (such as dopamine, GABA, 5-hydroxytryptamine, and norepinephrine) and amino acids. SGK3 is a potent regulator of CreaT (Shojaiefard et al., 2005) (Figure 4), which might regulate the creatine uptake. CreaT defect could lead to seizures, intellectual disability, and language disorders (Battini et al., 2007).

##### 1.4.6.2 Renal citrate transport

Citrate, known as citric acid, excreted into the urine can chelate with calcium, preventing calcium salt deposition, and thus inhibit the formation of urinary stones. The Na^+^ coupled dicarboxylate transporter 1 (NaDC1) participates in the renal tubular citrate transport. SGK3 could enhance the activity of NaDC1 (Figure 4), promoting the renal tubular reabsorption of citrate and the occurrence of calcium urolithiasis (Boehmer et al., 2004).

During the transportation of SGK3 with glucose, amino acids, and other substances, SGK3 enhances the activities of SGLT1 and SLC6A19 through the phosphorylation of Nedd4-1, promotes the absorption of glucose in the kidney and intestine, and regulates the transport of neutral amino acids in the kidney and intestine. SGK3 can up-regulate GluR1, GluR6, EAAT1, EAAT2, EAAT4, EAAT5, and SLC38A3, participate in the transport of glutamate and glutamine in the nervous system, and play a role in (pathological) physiological process of various nervous system diseases. SGK3 positively regulates CreaT and NaDC1 and promotes the uptake of creatine and the absorption of citric acid by renal tubules (Table 4).

## 2 Conclusion

In summary, this review highlights the function of SGK3 in calcium–phosphorus metabolism by regulating the (re)absorption of calcium and phosphate in the intestine and kidney and mineralization of the bone. We here describe that SGK3 involves in the generation of neuronal and muscle action potentials by influencing the activity of sodium and potassium channels and maintaining the cell electrical signal, excitability, volume, and fluid balance. The effects of SGK3 on the transport of glucose and amino acids can participate in the absorption of nutrients and is associated with a variety of metabolic diseases. Particular evidence points to its role in cell growth, proliferation, migration, and apoptosis, and SGK3 is closely related to the (patho) physiological processes of tumor growth, hair growth, nervous system diseases, and cardiovascular diseases. Additionally, SGK3 can be used as a prognostic biomarker and a novel therapeutic target in tumor growth.

It is suggested that the activated SGK3 can activate a large number of ion channels, carriers and receptors, and Na^+^/K^+^- ATPase, which involve a wide range of substance transport. It is also a very interesting topic to further explore how SGK3 regulates these molecular mechanisms. The following is a summary of mechanisms of SGK3 regulating activity.

S419DSGK3 can activate epithelial calcium channels TRPV5 and TRPV6. The key for SGK3 to activate TRPV5 is the presence of the PDZ domain containing scaffold protein NHERF2 that binds to the PDZ-binding motif on the carboxy-terminal tail of the channel. However, the deletion of the C-terminal PDZ-binding motif on TRPV6 will not affect the activation of TRPV6 by S419DSGK3. S419DSGK3 activates TRPV6 by enhancing its protein abundance at the cell surface (Böhmer et al., 2007).

SGK3 serine phosphorylation sites in GSK3 will inhibit GSK3 activity, thereby increasing renal Npt2a activity. SGK3 inhibits the ubiquitination degradation of Npt2b by Nedd4-2 through the phosphorylation inactivation of Nedd4-2, thereby increasing the activity of Npt2b (Palmada et al., 2004a; Bhandaru et al., 2011; Foller et al., 2011).

SGK3 may promote the transcription of STIM1 and STIM2 by regulating NF kB (Schmid et al., 2012).

In 293T cells, FLAG-SGK3 can pull down SERCA2b but SGK3 does not regulate the SERCA2b expression. ATPase activity is critical to SERCA2b. After GSK650394 inhibited SGK3 in EXE-R cells, the SERCA2b ATPase activity was significantly impaired. Therefore, SGK3 retains the function of SERCA2b (Wang et al., 2017).

PI (3.5) P2 can activate Kir2.2. The 45–78 amino acid residues at the C-terminal on Kir2.2 are very sensitive to SGK3. SGK3 phosphorylates S318 of PI (3.5) P2 to activate Kir2.2. SGK3 does not significantly affect the expression of Kir2.1 on the surface of *Xenopus laevis* oocytes, but the co-expression with Kir2.2 can increase Kir2.1 protein abundance at the cell surface, indicating that SGK3 can promote the transport of heterogeneous complexes composed of Kir2.1 and Kir2.2 channel proteins to the cell surface (Seebohm et al., 2012).

The PY motif on hERG is combined with the WW domain on Nedd4-2. SGK3 inactivates Nedd4-2 by phosphorylating Ser-444 (and Ser-338) residues in the Nedd4-2WW domain, thereby inhibiting the ubiquitination and internalization degradation of hERG by Nedd4-2, and finally increasing the Na channel abundance at the cell surface. According to the report, the GTP enzyme Rab11 could transport membrane proteins to the cell surface and interacted with hERG protein. SGK phosphorylates and activates PIKfyve, and PIKfyve interacts with phosphatidylinositol-3-phosphate to produce PI (3.5) P2. PIKfyve and its product PI (3.5) P2 can promote the Rab11-mediated insertion of the Kv7.1 channel protein into the plasma membrane. Similarly, SGK can also promote Rab11 to insert the recovered internalized hERG into the cell surface (Lamothe and Zhang., 2013).

In theory, SGK3 can change its activity by directly phosphorylating channel proteins or other signaling molecules. SGK3 can also inhibit the degradation of ubiquitinated target proteins by phosphorylating and inactivating Nedd4-2. However, it is unknown whether BKCa is targeted by Nedd4-2 (Ahmed et al., 2016b).

SGK3 can activate Na^+^/K^+^ ATPase, which may up-regulate K channels (Pao., 2012).

ENaC is composed of three subunits called α, β, and *γ* and the C-terminal of each subunit contains a proline-rich PPXY (PY) motif, wherein the C-terminal of α and *γ* subunits also contains the SGK consensus motif RXRXX (S/T). Ubiquitin-protein ligase Nedd4-2 recognizes the PY motif of ENaC and degrades ENaC through ubiquitinated ENaC. SGK3 inactivates Nedd4-2 by phosphorylating and inhibits Nedd4-2 to degrade ENaC. Because ENaC contains SGK3 phosphorylation consensus motifs, SGK3 is very likely to directly phosphorylate ENaC to affect its activity (Alexei Diakov and Korbmacher., 2004).

Unlike SGK1 and SGK2, the Phox-homologous domain at the end of SGK3 can locate SGK3 to the early endosome and this subcellular localization is important for the activation of NHE3 (Pao., 2012).

Nav1.5 contains S484 and S664 sites of the SGK3 phosphorylation consensus motif, which will change Nav1.5 gating properties. SGK3 can also inhibit the Nedd4-2 ubiquitination degradation of Nav1.5 through the phosphorylation inactivation of Nedd4-2 (Céline Marionneau et al., 2015).

SGK3 inhibits Nedd4-2 and then inhibits the ubiquitination degradation of CIC-Ka/barttin and CIC-2. In addition, SGK is still effective even if Nedd4-2 cannot interact with CIC-Ka/barttin. SGK may also affect CIC-Ka/barttin through other unknown mechanisms, for example, SGK3 directly phosphorylates the motif on CIC-Ka (Arg Val Arg Thr Thr-187Thr). CIC also contains SGK consensus sites (Ser82) (Embark et al., 2004b; Palmada et al., 2004b).

SGK3 phosphorylation inactivates Nedd4-2, which inhibits the ubiquitination clearance of SGLT1 in the cell membrane, thus increasing the abundance of the glucose transporter SGLT1 protein in the cell membrane (Dieter et al., 2004).

SGK3 can activate SLC6A19 by increasing the abundance of SLC6A19 proteins at the cell surface without changing the substrate affinity of SLC6A19 (Bohmer et al., 2010).

In *Xenopus laevis* oocytes, SGK3 may regulate the intermediate molecules of hASCT2 through phosphorylation or directly phosphorylating hASCT2 at non consensus sites, thus increasing the maximum transport rate without affecting the substrate affinity of hASCT2 to enhance the abundance of hASCT2 proteins at the cell surface and activate hASCT2 (Palmada et al., 2005).

SGK3 can increase GluR1 activity by increasing the abundance of GluR1 proteins at the cell surface. SGK3 can also partially up-regulate the GluR6 protein abundance in the cell membrane. However, GluR1 is more sensitive to SGK3, and GluR6 is more sensitive to SGK1 (Strutz-Seebohm et al., 2006).

SGK can directly phosphorylate the site on EAAT1 or inhibit Nedd4-2 to down regulate EAAT1 by phosphorylating and inactivating Nedd4-2. EAAT2 has no SGK consensus site. SGK3 improves the maximum transport rate and increases the abundance of EAAT2 proteins at the cell surface without affecting the affinity of EAAT2 for substrates (Boehmer et al., 2006).

SGK3 enhances glutamine-induced current by inhibiting Nedd4-2 and SN1 ubiquitination and degradation (Boehmer et al., 2003c).

SGK is unlikely to phosphorylate SLC6A8 directly. SGK3 can improve SLC6A8 activity by increasing the maximum transport rate of the carrier through a currently unknown protein (the protein itself targets the creatine transporter) (Shojaiefard et al., 2005).

SGK3 does not significantly change the concentration substrate affinity of the NaDC1 transporter but by an increase of its maximum transport rate to activate NaDC1 (Boehmer et al., 2004).

In the future, additional downstream target proteins as well as molecular and disease mechanisms will almost certainly be discovered in further research on SGK3, which will help in understanding the physiological and pathophysiological significance of SGK3 in detail and provide more pieces of evidence for this field.
